# Expression of human DNA mismatch-repair protein, hMSH2, in patients with oral lichen planus

**DOI:** 10.3892/etm.2014.2053

**Published:** 2014-11-06

**Authors:** HAO-BO LI, YING-HUAI ZHANG, HUI-ZHEN CHEN, YONG CHEN

**Affiliations:** 1 Oral Medicine Department, Second Hospital of Hebei Medical University, Shijiazhuang, Hebei 050000, P.R. China; 2 Oral Surgery Department, Central Hospital of Cangzhou, Cangzhou, Hebei 061000, P.R. China

**Keywords:** oral lichen planus, mismatch repair genes, hMSH2, immunohistochemistry

## Abstract

hMSH2 is one of the human DNA mismatch repair genes that plays an important role in reducing mutations and maintaining genomic stability. The aim of the present study was to detect the expression and significance of hMSH2 protein in patients with oral lichen planus (OLP). The expression levels of hMSH2 in the OLP group (n=51) and control group with normal oral mucosa (NM; n=40) were detected using an immunohistochemical method and subsequently assessed. The positive rate of hMSH2 expression in the OLP group was 52.94%, while the rate was 80% in the control group, exhibiting a statistically significant difference (χ^2^=7.1993; P<0.05). However, the expression of hMSH2 in the OLP tissues was not shown to significantly correlate with the patient gender, age and type of OLP (P>0.05). In conclusion, the protein expression levels of hMSH2 in the OLP tissues were significantly reduced as compared with that in the NM tissues, indicating that hMSH2 plays a role in the development of OLP. Therefore, hMSH2 may be used as a biomarker for evaluating the cancer risk of patients with OLP.

## Introduction

Mismatch repair (MMR) genes provide repair mechanisms for gene replication (1,2). These genes are not oncogenes or tumor suppressor genes, but an additional type of cancer-related gene. Recently, MMR genes have become increasingly studied to investigate the molecular mechanisms underlying the onset of cancer. The main function of MMR is to identify and repair the mismatched bases in base-specific DNA replication, in order to ensure the high-fidelity of DNA replication, control the occurrence of mutations and maintain the genomic stability (3). Currently, there are nine genes involved in MMR that encode a variety of MMR proteins, which specifically recognize and repair the mismatched bases (4,5). Mutations in any gene of this system can cause the dysfunction of MMR and result in genetic instability, presenting as replication errors or microsatellite instability, consequently leading to cancer. hMSH2 was the first human MMR gene to be isolated, whose mutations are the highest among the nine MMR genes (6). hMSH2 has been shown to recognize mismatched bases (7), and play an important role in reducing the number of mutations and maintaining the stability of genes.

Oral lichen planus (OLP) is a common non-infectious oral disease, with an incidence rate of 1–2% in the adult Swedish population (8). The disease is more common among individuals aged >40 years, particularly in females (9). The pathogenesis of OLP is multifactorial, and may include autoimmunity, chronic local irritation, mental factors, bacterial or viral infections and other factors (10,11). However, the exact mechanism is unknown. Although OLP is a benign disease, it does have the possibility of cancerization (12). Thus, OLP has been classified as a potentially malignant lesion by the World Health Organization (13). The incidence rate of OLP cancerization varies among different epidemiological surveys, ranging between 0.4 and 5.6%, and commonly between 1 and 2% (14,15). Thus, the cancer risk, influencing factors and cancerization mechanisms of OLP have attracted increasing attention.

In recent years, the single nucleotide database of hMSH2 has been established (16,17), which has resulted in increased research into the role of the hMSH2 gene in carcinogenesis. The expression of hMSH2 has been reported to change in oral squamous cell carcinoma (18); however, the expression of hMSH2 in OLP and its role in carcinogenesis has yet to be reported.

The aim of the present study was to investigate the role of hMSH2 in the pathogenesis and carcinogenesis of OLP by detecting the expression of hMSH2 in OLP tissues using immunohistochemistry. The results may provide novel ideas for accurately predicting the cancer risk of OLP and developing personalized treatment.

## Materials and methods

### Tissue samples

Formalin-fixed, paraffin-embedded, human OLP specimens were obtained from the Pathology Department at the Second Affiliated Hospital of Hebei Medical University (Shijiazhuang, China). The specimens included 24 cases of reticular type OLP and 27 cases of erosive/atrophy type OLP. The 51 patients comprising the OLP group included 22 males and 29 females, aged between 36 and 72 years (mean age, 53.54 years). Tissue samples were obtained during surgery, and none of the patients had received any treatment prior to surgery.

In total, 40 cases of normal oral mucosa (NM), obtained from the pericyst during cosmetics surgery of the alveolar bone, were used as a control group (NM group). The group included 22 males and 18 females, aged between 11 and 68 years (mean age, 31.30 years). The study was conducted in accordance with the Declaration of Helsinki and with approval from the Ethics Committee of Hebei Medical University. Written informed consent was obtained from all the participants.

### Immunohistochemical analysis

All immunohistochemical analyses were performed using horseradish peroxidase-labeled streptavidin. Tissue sections measuring 4–5 μm were mounted on Poly-L-lysine-coated slides (Sigma-Aldrich, St. Louis, MO, USA). Following deparaffinization using a standard protocol, sections were treated with 3% H_2_O_2_ in methanol for 25 min to block the endogenous peroxidase activity. The slides were subsequently microwaved in citrate buffer (pH 6.0) for 15 min at 92–98°C, and cooled to room temperature. After blocking with normal goat serum (Invitrogen Life Technologies, Carlsbad, CA, USA) for 30 min at 37°C, the slides were incubated with a polyclonal rabbit anti-human hMSH2 antibody [diluted 1:75 in phosphate-buffered saline (PBS); SC-22771; Santa Cruz Biotechnology, Dalla, TX, USA] overnight at 4°C in a high-humidity chamber. Oral squamous cell carcinoma (OSCC) tissue with known hMSH2 expression served as a positive control. For the negative control, PBS was used instead of a primary antibody. Subsequent steps were performed using an SP kit (Hebei Bohai Biology Co., Ltd.), according to the manufacturer’s instructions. Immunoreactivity was visualized using a solution of diaminobenzidine as the chromogen, and the nuclei were counterstained with hematoxylin.

### Assessment of staining

All the slides were interpreted by an experienced pathologist, according to the standards reported by Lo Muzio *et al* (18). Positive staining of hMSH2 was scored in the nucleus and in the plasma with brown particles. At least five randomly selected high-power fields (magnification, ×400) were examined. The proportion of immunoreactive cells in 100 cells per field was calculated, in which <5% was regarded to show negative expression (−), while ≥5% was considered to indicate positive expression (+).

### Statistical analysis

Statistical analysis was performed using the SPSS 17.0 statistical software program (SPSS, Inc., Chicago, IL, USA). The χ^2^ or Fisher’s exact tests were used to compare the differences between groups. Correlation analysis between the expression of hMSH2 and the clinical characteristics was performed using the χ^2^ test. For all analyses, P<0.05 was considered to indicate a statistically significant difference.

## Results

### hMSH2 expression

**Figure 1:**
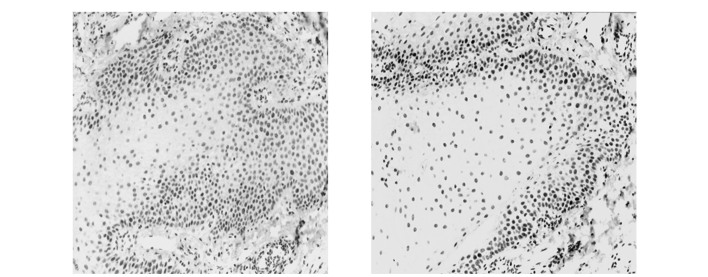
Expression of hMSH2 in normal mucosa (SP; magnification, ×400).

**Figure 2:**
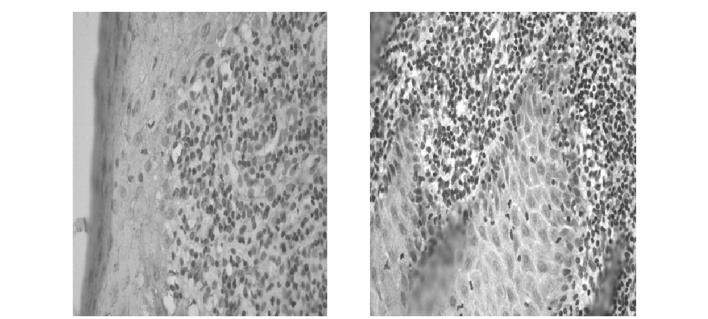
Expression of hMSH2 in patients with oral lichen planus (SP; magnification, ×400).

As shown in Table I, the positive rate of hMSH2 expression in the OLP tissues was 52.94%, which was significantly lower compared with that in the control group (80%; χ^2^=7.1993; P<0.05). In the NM tissue, hMSH2 expression was primarily observed in the cellular nuclei of the basal and spinous layer (Fig. 1). However, in the OLP tissues, positive staining of hMSH2 was predominantly observed in the cytoplasm (Fig. 2).

### Associations between hMSH2 expression and clinicopathological features

In the OLP tissues, the expression of hMSH2 was not found to significantly correlate with the gender or age of the patient, or the type of OLP (P>0.05; Table II).

## Discussion

OLP is a common non-infectious disease of the oral mucosa; however, the pathogenesis remains unknown. Currently, OLP is considered to be a potential malignant lesion, and the majority of studies hypothesize that the condition may develop into OSCC (19,20). However, whether OLP exhibits cancerization remains controversial (21), with certain studies considering OLP to be more similar to other benign oral mucosal lesions, and pose no higher risk of cancerization (22,23). The aim of the present study was to observe the protein expression of hMSH2 in the MMR system of OLP, in order to investigate the role of hMSH2 in the occurrence and development of OLP and assess the cancerization trend of OLP.

The MMR system is a basic biological mechanism for maintaining the high fidelity of DNA replication and the stability of the genetic material by controlling genetic mutations. The system is an important barrier to prevent the onset of tumors. MMR proteins always exists in multimeric complex forms to recognize and specifically repair the mismatched bases during DNA replication, and may subsequently induce the apoptosis of cells with severely damaged DNA (24). The inactivation of MMR genes may present as the activation of oncogenes or the inactivation of tumor suppressor genes caused by microsatellite instability, or directly causing mutations in oncogenes or tumor suppressor genes, thereby inducing carcinogenesis (25,26).

To date, nine human MMR genes have been identified, including hMSH2, hMSH6, hMSH5, hMSH4, hMSH3, hMLH1, hPMS1, hPMS2 and hMLH3 (4). hMSH2 was the first human MMR gene to be isolated, which was isolated from human hereditary nonpolyposis colorectal cancer (HNPCC). A number of studies have confirmed that gene mutations of hMSH2 play crucial roles not only in the pathogenesis of HNPCC (27), but also in the pathogenesis of a variety of other tumors (28–31), including cancer of the endometrium, rectal cancer, ovarian cancer, bladder cancer, as well as OSCC (18). Generally, stronger expression of the MMR proteins can lead to a stronger repairing ability. Thus, the positive rate of hMSH2 in non-tumor tissues is significantly higher compared with that in tumor tissues. For example, the expression of hMSH2 protein in HNPCC, cervical squamous cell carcinoma and prostate cancer is significantly lower compared with that in normal control tissues (32). Lo Muzio *et al* (18) analyzed the expression of hMSH2 in OSCC and normal oral mucosal tissues using immunohistochemical methods, and found that the deletion of hMSH2 protein may be a potential indicator for OSCC.

In the present study, the positive rate of hMSH2 expression in the OLP tissue was found to be significantly lower compared with that in the control group. Thus, it was hypothesized that defects of MMR function may play certain roles in the occurrence and development of OLP, supporting the hypothesis that OLP is a potentially malignant lesion that poses a cancer risk. Pimenta *et al* (33) also examined the expression of hMSH2 protein in 26 cases of OLP (12 cases of reticular type and 14 cases of atrophic or erosive type) using immunohistochemistry. The authors found that the positive rate of hMSH2 expression was 46.54% in reticular type OLP, 48.79% in atrophic or erosive type OLP and 61.29% in the normal mucosa, which is consistent with the observations of the present study.

In conclusion, the present study demonstrated that hMSH2 expression was significantly reduced in OLP tissues, resulting in MMR defects in the cells, which may subsequently cause genetic instability and lead to the onset of OSCC. However, the exact mechanisms of hMSH2 in the development and carcinogenesis of OLP should be confirmed by further studies.

## Figures and Tables

**Table I:** Expression of hMSH2 in patients with NM and OLP.

		hMSH2 (n)		
			
Groups	Cases (n)	+	−	Positive rate (%)
NM	40	32	8	80.0
OLP	51	27	24	52.94^a^

a P<0.05, vs. NM.

NM, normal mucosa; OLP, oral lichen planus.

**Table II:** Association between hMSH2 expression and clinicopathological features of patients with OLP.

		hMSH2 (n)				
					
Parameters	Cases (n)	+	−	Positive rate (%)	χ^2^	P-value
Gender					0.04	
Male	22	12	10	54.55		>0.05
Female	29	15	14	51.72		
Age (years)					0.14	
<50	14	8	6	57.14		>0.05
≥50	37	19	18	51.35		
Types					0.92	
Reticular	24	11	13	45.83		>0.05
Erosive/Atrophic	27	16	11	59.26		

OLP, oral lichen planus.
